# Remnant cholesterol inflammation index as a predictor of mortality in patients with acute decompensated heart failure: evidence from the Jiangxi, China cohort

**DOI:** 10.3389/fendo.2026.1792583

**Published:** 2026-04-23

**Authors:** Guoan Jian, Zhenyu Wang, Juan Wang, Houhui Lan, Kun Jiang, Zihao Lu, Guotai Sheng, Guobo Xie, Wei Wang, Yang Zou, Chunyuan Jiang

**Affiliations:** 1 Jiangxi Cardiovascular Research Institute, Jiangxi Provincial People’s Hospital, The First Affiliated Hospital of Nanchang Medical College, Nanchang, Jiangxi, China; 2 Jiangxi Medical College, Nanchang University, Nanchang, Jiangxi, China; 3 Department of Cardiology, Jiangxi Provincial People’s Hospital, The First Affiliated Hospital of Nanchang Medical College, Nanchang, Jiangxi, China

**Keywords:** acute decompensated heart failure, cohort, heart failure, mortality, remnant cholesterol inflammation index

## Abstract

**Introduction:**

The Remnant Cholesterol Inflammation Index (RCII) is a novel composite biomarker that integrates atherosclerotic and inflammatory statuses. The present study aimed to investigate the association between RCII and short-term prognosis in patients with acute decompensated heart failure (ADHF).

**Methods:**

A total of 1,055 ADHF patients enrolled in the Jiangxi-ADHF II cohort (2018–2024) were included. Multivariable Cox regression and restricted cubic spline analyses were used to assess the association between RCII and 30-day mortality in ADHF patients. Threshold analysis was performed to identify a potential inflection point. The DeLong test was applied to evaluate the incremental predictive value of RCII over RC and traditional lipid parameters. Furthermore, based on baseline characteristics, a baseline risk model for predicting short-term prognosis of ADHF was established, and the incremental predictive value of adding RCII to this model was evaluated. Mediation analysis examined the potential mediating effects of gamma-glutamyl transferase and albumin.

**Results:**

During the 30-day follow-up, 85 death events (8.06%) were recorded. After adjustment for multiple confounders, a higher RCII level was significantly associated with an increased risk of 30-day mortality in ADHF patients. Exploratory restricted cubic spline analysis further revealed an inverted L‑shaped dose–response relationship between LnRCII and 30‑day mortality (*P* for nonlinearity=0.049). Further predictive analyses demonstrated that RCII significantly outperformed RC and traditional lipid parameters in predicting short-term mortality in ADHF patients. Adding RCII to the baseline risk model significantly improved the C-index from 0.85 to 0.87 (*P* < 0.01), with an integrated discrimination improvement of 0.05 (*P* = 0.04). Mediation analysis indicated that albumin partially mediated the RCII–mortality association (the mediated proportion was approximately 11.45%).

**Discussion:**

Our analysis identified a higher RCII as a significant predictor of increased short-term mortality in patients with ADHF, establishing RCII as a novel and important predictor of poor short-term prognosis in ADHF.

## Graphical Abstract

**Figure:**
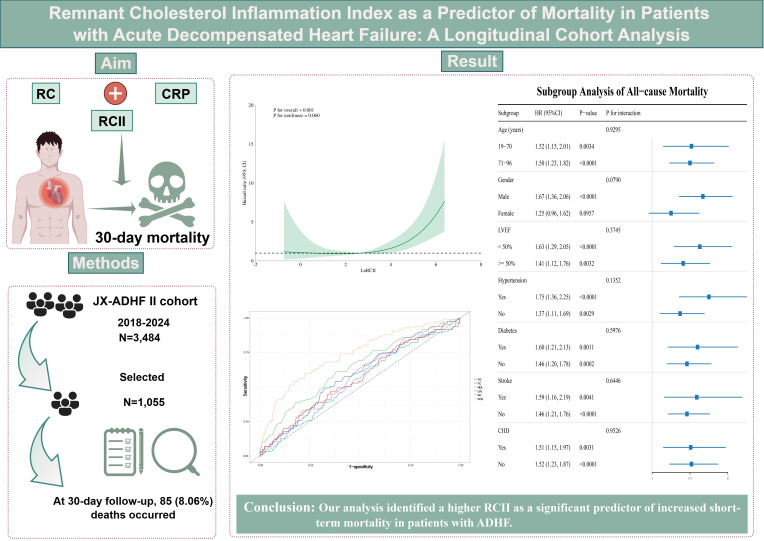

## Background

Acute decompensated heart failure (ADHF) is a life-threatening clinical syndrome that occurs in the terminal stage of cardiovascular disease (CVD) progression and usually requires urgent hospitalization for intervention (1, 2). In China, ADHF has become a severe public health challenge. Epidemiological data indicate that the annual number of hospitalizations for ADHF in China exceeds 10 million, with approximately 40% of patients requiring multiple (≥3 times) readmissions within one year due to recurrent exacerbations of HF and an annual medical expenditure of approximately $4,000 per patient (3, 4). In recent years, despite advances in management strategies such as pharmacotherapy and mechanical circulatory support, the clinical prognosis of ADHF patients remains unsatisfactory (5, 6). Studies have shown that approximately 25% of patients are readmitted within 30 days after discharge due to recurrent HF, with a mortality rate of around 10% during the same period (7–9). This not only imposes a significant financial burden on patients and their families but also places a heavy strain on the healthcare system.

Atherosclerosis represents a crucial risk factor for various CVDs including HF (10–12), and its development and progression are primarily driven by dysregulated lipid metabolism and inflammatory responses (13, 14). Evidence has demonstrated that maintaining low-density lipoprotein cholesterol (LDL-C) within the optimal range can effectively reduce cardiovascular risks. However, a subset of patients still exhibits significant residual risk in clinical settings, which suggests that pathogenic factors independent of LDL-C exist (15–17). Recent studies have indicated that elevated levels of remnant cholesterol (RC) and low-grade inflammation, primarily characterized by C-reactive protein (CRP), are major contributors to this residual cardiovascular risk (18–21). RC is defined as the sum of cholesterol carried by all triglyceride (TG)-rich lipoproteins and their metabolic remnants, consisting primarily of cholesterol from very-low-density lipoproteins and intermediate-density lipoproteins under fasting conditions, with chylomicron remnants also contributing in the non-fasting state (22, 23). RC plays a significant role in the pathological progression of atherosclerosis by inducing vascular endothelial dysfunction, promoting lipid deposition beneath the vascular endothelium, and accelerating foam cell formation (24, 25). During this process, various inflammatory cells are activated, leading to a chronic low-grade inflammatory state both locally and systemically (26, 27). Observational and genetic studies have further confirmed that RC itself exhibits pro-inflammatory properties, and elevated RC levels are often accompanied by increased CRP levels (28, 29). Therefore, the combination of elevated RC and low-grade inflammation may synergistically drive the progression of cardiovascular events.

Mounting recent evidence shows that individuals with concurrent elevations in both RC and CRP face a significantly higher risk of cardiovascular events and adverse outcomes compared with those with an elevation in either marker alone (30–34). This indicates that the combined assessment of RC and CRP holds promise for enabling more precise cardiovascular risk stratification. Building upon this theoretical foundation, a composite index that simultaneously reflects lipid and inflammatory status, termed the Remnant Cholesterol Inflammation Index (RCII), may therefore carry significant clinical value. However, current research on RCII remains relatively limited. Existing evidence has primarily focused on assessing cardiovascular risk and all-cause mortality risk in populations with prediabetes and diabetes, as well as in predicting stroke and all-cause mortality risk in the general population (35–37). The value of RCII for prognostic evaluation in high-risk populations such as those with ADHF remains unknown. Therefore, this study aims to investigate the impact of RCII on 30-day all-cause mortality in patients with ADHF, to provide new evidence-based insights for risk stratification and prognostic assessment in this population.

## Methods

### Study population

The Jiangxi-ADHF II is a retrospective cohort study conducted at Jiangxi Provincial. It aimed to integrate in-hospital clinical data and post-discharge follow-up data from patients with ADHF, with the goal of identifying effective early risk stratification methods and generating evidence-based insights to better guide clinical practice and improve patient outcomes. The Jiangxi-ADHF II cohort consecutively enrolled 3,484 patients with ADHF who were admitted to Jiangxi Provincial People’s Hospital between January 2018 and January 2024. An episode of ADHF was defined as the development of new or worsening signs and symptoms on the basis of chronic HF, typically resulting in hospitalization or intensive emergency treatment (1, 2). This study strictly adhered to the latest European Society of Cardiology or American College of Cardiology/American Heart Association heart failure guidelines current during the enrollment period for patient screening. All enrolled patients were required to present worsening clinical symptoms along with corresponding physical signs of heart failure and objective examination evidence. The specific diagnostic criteria were as follows:

(I) Worsening clinical symptoms (at least 1 item required):

1. Respiratory dysfunction: including exertional dyspnea, paroxysmal nocturnal dyspnea, or orthopnea;
2. Systemic congestion: such as peripheral edema, hepatic congestion, or ascites;
3. Signs of tissue hypoperfusion: such as oliguria/anuria, cold extremities, altered mental status, hyperlactatemia, or metabolic acidosis.

(II) Objective evidence of heart failure (at least 1 item required):

1. Imaging evidence: pulmonary edema indicated by physical examination or chest radiograph;
2. Biomarkers: elevated levels of B-type natriuretic peptide or N-terminal pro-B-type natriuretic peptide (NT-proBNP);
3. Structural or functional abnormalities: cardiac structural or functional abnormalities confirmed by echocardiography.

In the current study, we excluded participants with the following characteristics: (1) those diagnosed with uremia, chronic kidney disease on hemodialysis, or liver cirrhosis (n=231 + 42) to minimize confounding from non-cardiac causes of fluid and sodium retention; (2) those with a malignancy (n=160), due to its significant impact on mortality; (3) those who had undergone percutaneous coronary intervention within the past 3 months (n=102) to avoid the impact of reperfusion therapy on short-term prognosis; (4) those with implanted pacemakers (n=121) to avoid potential interference with autonomic function assessment; (5) those who were minors (n=22) or pregnant (n=4); (6) those with missing RC data (n=437); (7) those with missing CRP data (n=1,310). Ultimately, a total of 1,055 subjects were included in this study. Given the high proportion of missing data for RC and CRP, we conducted an exploratory analysis of the missingness pattern of RCII data. To evaluate the pattern of missing data, we compared the key demographic characteristics, clinical features, and incidence of study endpoints between the RCII complete-case group and the group with missing RCII data. The analysis showed similar distribution s between the two groups in terms of baseline characteristics and study endpoints (**Supplementary Table 1**; most *P* > 0.05). These results suggest that the RCII data were missing at random.

### Ethical approval

The design and implementation of this study were strictly performed in accordance with the ethical principles of the Declaration of Helsinki and were approved by the Ethics Review Committee of Jiangxi Provincial People’s Hospital (Ethical approval number: 2024-01). The reporting of the study results rigorously follows the Strengthening the Reporting of Observational Studies in Epidemiology guidelines to ensure transparency and scientific rigor.

### Data collection

Trained medical personnel collected patient data from electronic medical records upon admission. The collected data included demographic information (sex, age), comorbidities (hypertension, diabetes, coronary heart disease [CHD], stroke), New York Heart Association (NYHA) functional classification, lifestyle habits (smoking, drinking), and echocardiographic parameters (left ventricular ejection fraction: LVEF). It should be noted that the diagnosis of comorbidities was based on the patients’ past medical history and medication records.

Venous blood samples were collected from patients within 24 hours of admission and sent to the Clinical Laboratory Center of Jiangxi Provincial People’s Hospital for analysis. The measured parameters included: complete blood count parameters—red blood cell count, white blood cell count, and platelet count; liver function indices—albumin (ALB), alanine aminotransferase (ALT), aspartate aminotransferase (AST), and gamma-glutamyl transferase (GGT); renal function indices—creatinine (Cr), blood urea nitrogen (BUN), and uric acid (UA); lipid profile—total cholesterol (TC), TG, LDL-C, and high-density lipoprotein cholesterol (HDL-C); glucose parameter—fasting plasma glucose (FPG); and cardiac function parameter— NT-proBNP. Specifically, blood samples for assessing FPG, liver function (ALT, AST, GGT), and lipid metabolism (TC, TG, LDL-C, HDL-C) were collected after a strict fast of at least 8 hours (upon admission or the following morning), to minimize the influence of dietary factors on the accuracy of the results. Of note, among the ADHF patients at admission, 2.94% had a systolic blood pressure below 90 mmHg, 14.22% had a diastolic blood pressure below 60 mmHg, and 7.49% had a CRP level exceeding 10 mg/dL. These categorical data indicate that a small proportion of patients had hemodynamic instability or acute infection at the time of blood sample collection.

### Calculation of RCII

RC (mg/dL) = TC(mg/dL) – HDL-C(mg/dL) – LDL-C(mg/dL).

RCII = RC (mg/dL) *CRP(mg/dL) (37). RCII was analyzed as a continuous variable in this study. Its frequency distribution histogram (**Supplementary Figure 1**) exhibited a right-skewed pattern. To meet the normality assumption for parametric statistical analyses, we utilized the natural logarithm-transformed value of RCII (LnRCII) in subsequent analyses.

### Study outcome

The primary outcome of this study was all-cause mortality within 30 days after admission in patients with ADHF. The observation period commenced at the time of hospital admission. Information on patient survival status was collected and verified by uniformly trained study personnel through multiple approaches. In-hospital mortality was determined by reviewing hospital medical records, with the cause of death documented. Out-of-hospital mortality was confirmed via telephone, text message, or outpatient follow-up. The cause of death was independently determined using discharge summaries and information on the circumstances of death obtained from family members, and was classified as cardiac death or non-cardiac death.

### Assessment and handling of missing data

The extent of missing data in this study is detailed in the supplementary materials (**Supplementary Table 2**, **Supplementary Figure 2**). The maximum number of missing observations for any variable was 42. Analysis of the missing data patterns (**Supplementary Figure 3**) showed that the liver function indices (ALT, AST, GGT, ALB), renal function indices (Cr, BUN), and UA had significant co-occurrence of missingness. This pattern suggested that the mechanism for these missing values was consistent with the Missing at Random assumption. In addition, the missing patterns of LVEF and FPG were independent of the missing patterns of other covariates, suggesting characteristics consistent with missing completely at random. To further assess the interdependencies of missingness among variables, we constructed a correlation matrix plot of missingness indicator variables (**Supplementary Figure 4**), and the results demonstrated a high consistency with the missing patterns shown in **Supplementary Figure 3**. Given the overall low proportion of missing data and the fact that the primary variables met the missing at random assumption, the original dataset was retained for analysis in this study, with complete-case analysis serving as the foundational statistical approach.

### Statistical analysis

Participants were stratified into four groups (Q1-Q4) based on the quartiles of RCII. Quantitative data were presented as mean ± standard deviation or median (interquartile range); categorical variables were presented as frequencies (percentages). Inter-group differences were compared using one-way analysis of variance, the Kruskal-Wallis test, or the chi-square test, as appropriate.

The 30-day survival curves for patients in different RCII groups were plotted using the Kaplan-Meier method, and the survival differences between groups were assessed using the log-rank test. A multivariable-adjusted Cox proportional hazards model was employed to determine the association between RCII and 30-day mortality in ADHF patients, with results expressed as hazard ratios (HRs) and 95% confidence intervals (CIs). Before model construction, the proportional hazards assumption was validated using Schoenfeld residual tests (**Supplementary Table 3**) and residual plots (**Supplementary Figure 5**). The results indicated that all covariates satisfied the proportional hazards assumption (Global Schoenfeld Test p = 0.807). To avoid the impact of multicollinearity on model stability, the components of RCII, namely TC, HDL-C, LDL-C, and CRP, were excluded from the multivariable model. Given the strong correlations between TG and RC, as well as between FPG and diabetes, these variables were also excluded from the multivariable model. Additionally, we calculated the variance inflation factor of each variable (see **Supplementary Table 4**). Subsequently, three sequentially adjusted Cox regression models were constructed: Model I was adjusted for demographic characteristics (sex, age) and lifestyle factors (drinking status, smoking status); Model II further incorporated clinical comorbidities (hypertension, diabetes, stroke, CHD) and cardiac function indicators (NYHA functional classification, LVEF); Model III additionally included laboratory parameters (ALB, AST, GGT, Cr, BUN, UA, NT-proBNP) based on Model II. Furthermore, to explore the potential nonlinear relationship between LnRCII and the endpoint event, restricted cubic spline regression was used for fitting and visualization. When the results of the likelihood ratio test indicated the presence of a potential non-linear association, a two-piecewise Cox regression model was employed to identify the inflection point, with its specific value calculated through a recursive algorithm.

Exploratory subgroup analyses were performed to evaluate the potential effect modification of age, sex, LVEF, hypertension, diabetes, stroke, and CHD on the association between LnRCII and 30-day mortality in ADHF patients. Likelihood ratio tests were applied to quantify the interaction effects between LnRCII and each stratification variable.

To systematically compare the predictive performance of RCII with that of RC or traditional lipid parameters (TG, TC, HDL-C, LDL-C) for the prognosis of ADHF patients, we conducted receiver operating characteristic curve analyses, and calculated the corresponding area under the curve (AUC), optimal cut-off values, as well as sensitivity and specificity. DeLong’s test was employed for statistical comparison of AUC values among different predictive models. To investigate the incremental clinical value of RCII, we added it to a baseline risk model that included variables such as sex, age, alcohol consumption history, smoking history, hypertension, diabetes, stroke, CHD, NYHA classification, LVEF, ALB, AST, GGT, Cr, BUN, UA, and NT-proBNP, and evaluated the improvement in model predictive performance. The evaluation metrics included the continuous C index and the integrated discrimination improvement.

Furthermore, based on evidence from previous studies, we constructed mediation models to explore the potential mediating roles of the oxidative stress marker GGT (38) and the nutritional status indicator ALB (39) in the association between LnRCII and 30-day mortality in ADHF patients.

Finally, to evaluate the robustness of the study results, we performed a series of sensitivity analyses: (1) We excluded the frail subgroup (defined as patients with three or more comorbidities), given that frailty could influence adverse outcomes. (2) We also excluded patients with concurrent pulmonary infections at admission to reduce interference from acute inflammation. (3) We excluded patients on statin therapy at admission to minimize its potential effect on LnRCII. (4) We excluded patients who died within 48 hours of admission to mitigate reverse causality. (5) We repeated the primary analysis after constructing a complete dataset using mean or median imputation.

## Results

### Baseline characteristics stratified by RCII groups

**Figure 1:**
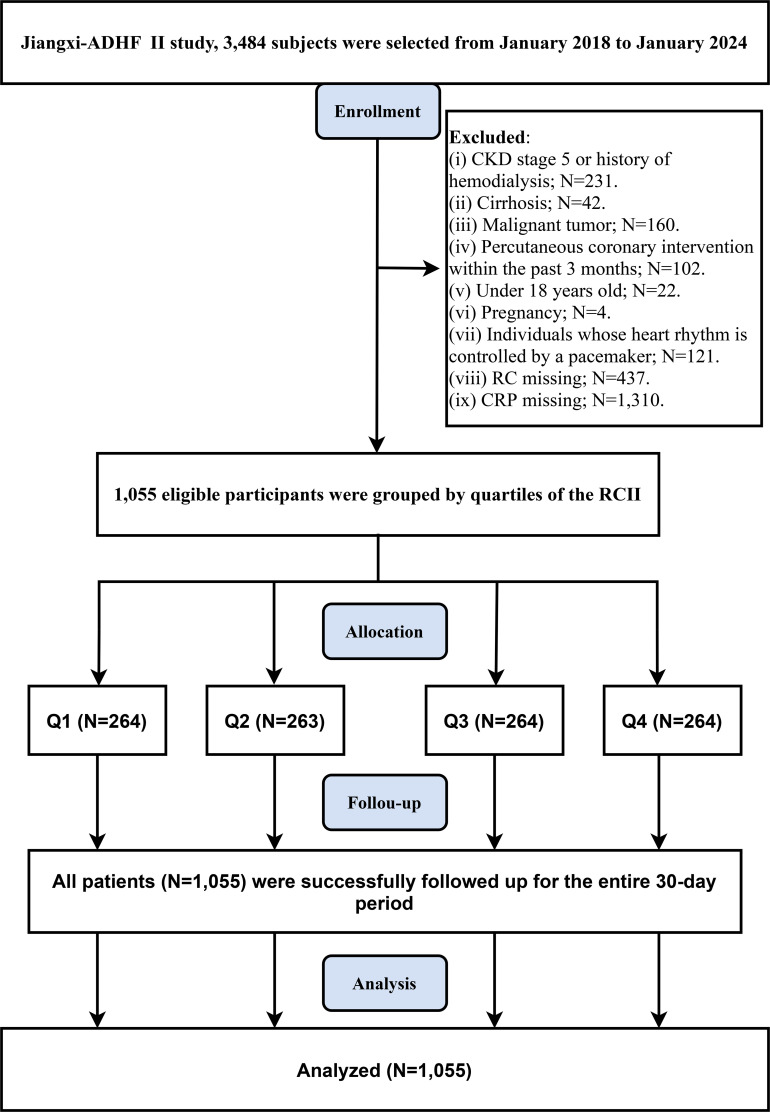
Flow chart of study participants.

The screening process of the study population is illustrated in **Figure 1**. This study ultimately included 1,055 patients with ADHF, with a mean age of 69 years, of whom 57.91% (n=611) were male. The baseline characteristics stratified by RCII quartiles are presented in **Table 1**. Briefly, compared with patients in the lowest quartile (Q1), those in the highest quartile (Q4) were generally older, more likely to be male, and had poorer cardiac function, a higher prevalence of alcohol consumption, and a greater comorbidity burden, including hypertension, diabetes, and CHD. In addition, regarding laboratory parameters, patients in the Q4 group exhibited higher levels of Cr, BUN, UA, TC, TG, RC, CRP, FPG, and NT-proBNP, along with lower levels of ALB and HDL-C. However, there were no significant between-group differences in the prevalence of stroke, drinking status, as well as LVEF, ALT, AST, GGT, and LDL-C.

**Table 1:** Summary of baseline characteristics of the study population according to RCII quartile group.

Variable	RCII quartiles				*P*-value
	Q1	Q2	Q3	Q4	
No. of subjects	264	263	264	264	
Age (years)	69.00 (58.00-78.00)	70.00 (59.00-79.00)	71.00 (61.00-81.00)	74.00 (62.75-81.00)	0.003
Gender					0.002
Male	145 (54.92%)	136 (51.71%)	152 (57.58%)	178 (67.42%)	
Female	119 (45.08%)	127 (48.29%)	112 (42.42%)	86 (32.58%)	
NYHA classification (n,%)					<0.001
III	203 (76.89%)	167 (63.50%)	161 (60.98%)	161 (60.98%)	
IV	61 (23.11%)	96 (36.50%)	103 (39.02%)	103 (39.02%)	
Hypertension					0.036
No	163 (61.74%)	145 (55.13%)	130 (49.24%)	150 (56.82%)	
Yes	101 (38.26%)	118 (44.87%)	134 (50.76%)	114 (43.18%)	
Diabetes					<0.001
No	220 (83.33%)	200 (76.05%)	175 (66.29%)	179 (67.80%)	
Yes	44 (16.67%)	63 (23.95%)	89 (33.71%)	85 (32.20%)	
Stroke					0.582
No	221 (83.71%)	217 (82.51%)	210 (79.55%)	212 (80.30%)	
Yes	43 (16.29%)	46 (17.49%)	54 (20.45%)	52 (19.70%)	
CHD					0.035
No	199 (75.38%)	187 (71.10%)	177 (67.05%)	170 (64.39%)	
Yes	65 (24.62%)	76 (28.90%)	87 (32.95%)	94 (35.61%)	
Drinking status					0.657
No	236 (89.39%)	243 (92.40%)	242 (91.67%)	241 (91.29%)	
Yes	28 (10.61%)	20 (7.60%)	22 (8.33%)	23 (8.71%)	
Smoking status					0.008
No	224 (84.85%)	231 (87.83%)	227 (85.98%)	205 (77.65%)	
Yes	40 (15.15%)	32 (12.17%)	37 (14.02%)	59 (22.35%)	
LVEF (%)	49.00 (39.00-57.75)	49.00 (37.00-56.00)	47.00 (39.00-56.00)	50.00 (40.00-57.00)	0.553
ALB (g/L)	36.66 (4.58)	35.42 (4.45)	34.74 (5.31)	32.53 (5.55)	<0.001
ALT (U/L)	21.00 (14.00-35.00)	21.50 (14.00-38.25)	23.00 (14.00-43.75)	21.00 (13.00-40.00)	0.586
AST (U/L)	25.00 (20.50-36.00)	25.00 (19.00-37.00)	29.00 (20.00-47.00)	27.50 (19.00-46.25)	0.066
GGT (U/L)	41.00 (23.00-69.00)	44.00 (25.75-77.50)	45.50 (27.00-93.00)	43.00 (27.00-76.00)	0.070
Cr (umol/L)	78.00 (63.00-101.25)	84.50 (68.00-115.50)	94.00 (73.00-130.00)	101.00 (75.00-155.00)	<0.001
BUN (mmol/L)	6.62 (5.06-8.66)	6.88 (5.34-9.24)	7.87 (6.00-10.94)	8.63 (6.43-13.65)	<0.001
UA (umol/L)	400.00 (312.75-481.50)	422.00 (339.00-503.00)	455.00 (339.00-567.00)	426.00 (315.00-542.50)	0.008
TC (mg/dL)	141.88 (35.53)	147.77 (36.26)	153.18 (42.05)	151.00 (51.97)	0.013
TG (mg/dL)	87.71 (69.99-118.06)	101.00 (77.97-138.66)	108.53 (83.73-144.20)	110.31 (83.28-151.51)	<0.001
HDL-C (mg/dL)	40.80 (33.64-47.56)	37.12 (31.13-45.05)	37.12 (30.07-46.02)	35.58 (27.75-43.70)	<0.001
LDL-C (mg/dL)	83.14 (65.35-106.63)	87.01 (68.64-112.34)	86.23 (69.90-107.21)	80.43 (64.10-103.73)	0.149
RC (mg/dL)	11.99 (6.96-17.40)	15.85 (10.83-23.01)	20.88 (14.31-30.26)	21.66 (15.85-30.55)	<0.001
CRP (mg/dL)	0.21 (0.13-0.31)	0.51 (0.40-0.88)	1.25 (0.78-1.90)	7.21 (3.79-10.70)	<0.001
FPG (mmol/L)	5.20 (4.60-6.00)	5.20 (4.60-6.00)	5.40 (4.70-6.30)	5.80 (4.80-6.90)	<0.001
NT-proBNP (pmol/L)	2919.50 (1516.50-4940.50)	3592.00 (1842.50-6984.00)	3750.50 (1763.25-7189.75)	4184.50 (1795.75-6949.50)	<0.001

CHD, coronary heart disease; NYHA, New York Heart Association; LVEF, left ventricular ejection fraction; TG, triglyceride; TC, total cholesterol; HDL-C, high-density lipoprotein cholesterol; LDL-C, low-density lipid cholesterol; Cr, creatinine; BUN, blood urea nitrogen; UA, uric acid; ALT, alanine aminotransferase; AST, aspartate aminotransferase; GGT, gamma-glutamyltransferase; ALB, albumin; NT-proBNP, N-Terminal Pro-Brain Natriuretic Peptide; BUN, urea nitrogen; FPG, fasting plasma glucose; RC, remnant cholesterol; RCII, remnant cholesterol inflammatory index.

### Follow-up outcomes

**Figure 2:**
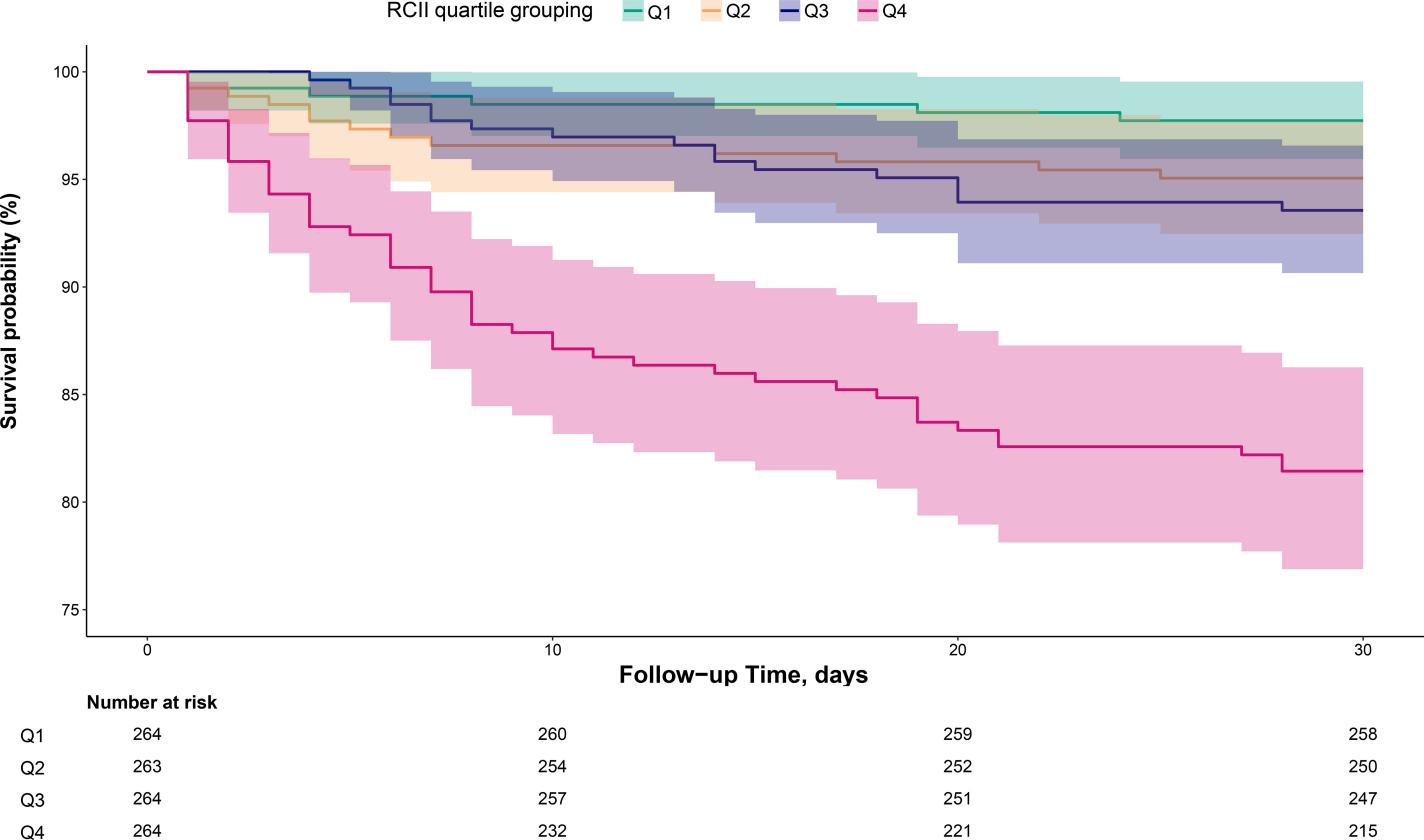
Cumulative survival rate curves of ADHF patients in the RCII group. RCII, Remnant Cholesterol Inflammation Index; ADHF, acute decompensated heart failure.

During the 30-day follow-up period, a total of 85 (8.06%) all-cause mortality events were observed, of which 66 (6.26%) were due to cardiovascular causes. As shown in **Figure 2**, the Kaplan-Meier survival curves stratified by RCII quartiles demonstrated that patients in the highest quartile had a significantly higher 30-day mortality rate than those in the lowest quartile (Log-rank *p* < 0.0001).

### Association between RCII and 30-day mortality in ADHF patients

**Table 2** presents the HRs for the association between RCII and 30-day mortality in ADHF patients. The results indicated that LnRCII consistently showed a significant positive correlation with 30-day mortality among ADHF patients across all models (*P* for trend < 0.001). Specifically, the HR associated with LnRCII showed a slight decrease with progressive model adjustment, although the positive association remained statistically significant throughout. In the fully adjusted Model III, each 1-unit increase in LnRCII (as a continuous variable) was associated with a 52% higher risk of 30-day mortality in ADHF patients (HR: 1.52, 95% CI: 1.28–1.79). When RCII was analyzed as a categorical variable, patients in the highest quartile (Q4) had a 240% increased risk of 30-day mortality compared with those in the lowest quartile (Q1) (HR: 3.40, 95% CI: 1.41–8.24).

**Table 2:** Multivariable Cox regression analysis of the association between RCII and 30-day mortality in patients with ADHF.

Independent variable	Hazard ratios (95% confidence interval)			
	Unadjusted model	Model I	Model II	Model III
LnRCII	1.72 (1.50, 1.97)	1.70 (1.48, 1.95)	1.63 (1.40, 1.90)	1.52 (1.28, 1.79)
RCII (quartiles)				
Q1	Ref	Ref	Ref	Ref
Q2	2.21 (0.84, 5.81)	2.14 (0.81, 5.63)	1.62 (0.61, 4.32)	1.01 (0.36, 2.80)
Q3	2.86 (1.13, 7.26)	2.70 (1.06, 6.85)	1.80 (0.70, 4.65)	1.13 (0.43, 3.00)
Q4	8.91 (3.82, 20.80)	7.92 (3.37, 18.59)	5.58 (2.35, 13.27)	3.40 (1.41, 8.24)
*P*-trend	<0.0001	<0.0001	<0.0001	0.0001

ADHF, acute decompensated heart failure; RCII, remnant cholesterol inflammatory index.

Model I adjusted for gender, age, drinking status, smoking status.

Model II adjusted for model I + hypertension, diabetes, stroke, CHD, NYHA classification, LVEF.

Model III adjusted for: Model II + ALB, AST, GGT, Cr, BUN, UA, NT-proBNP.

### Exploratory analysis of the dose-response relationship between LnRCII and 30-day mortality in ADHF patients

**Figure 3:**
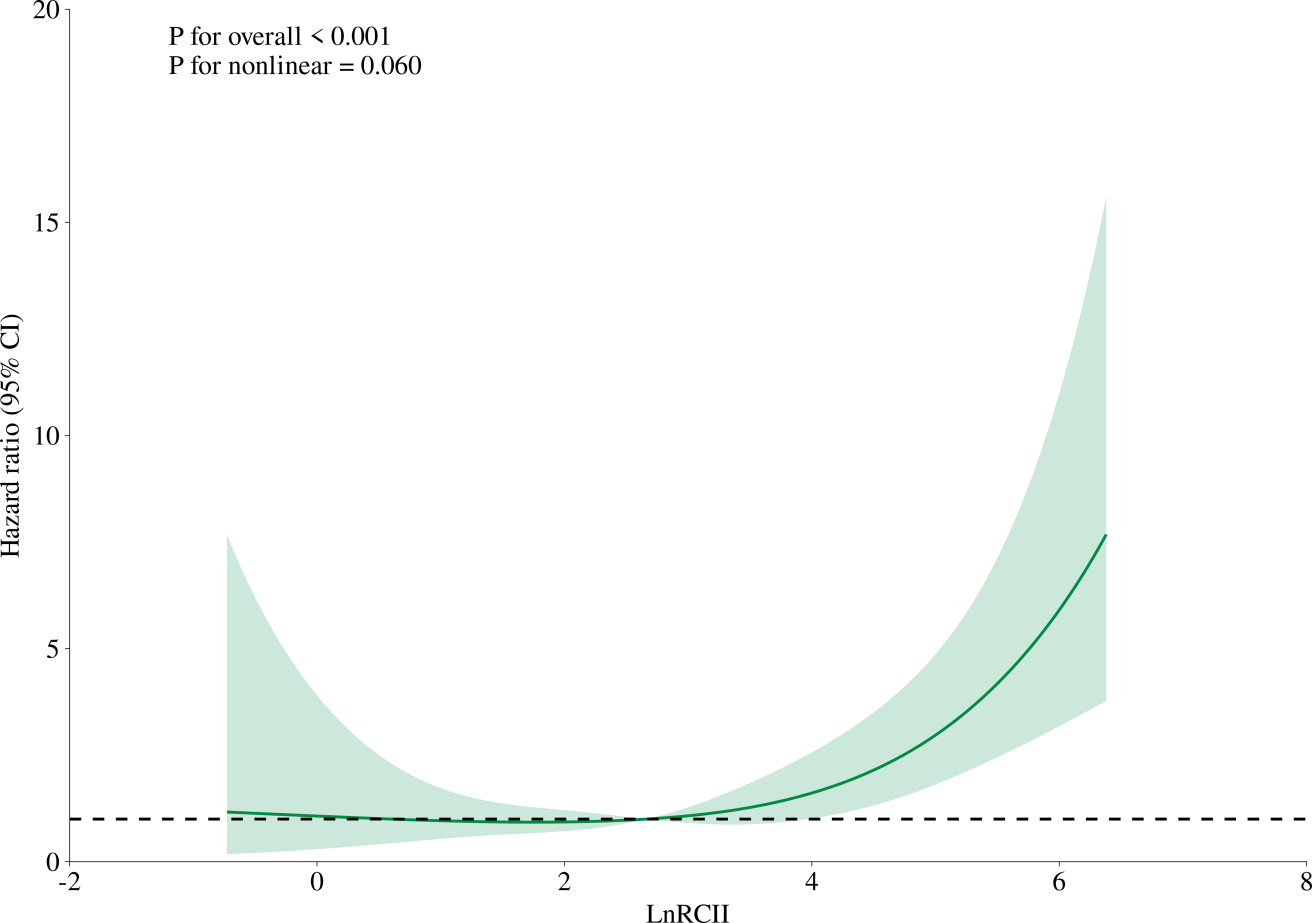
Fitting the dose-response relationship between RCII and 30-day mortality in ADHF patients with 4 knots restricted cubic spline. RCII, Remnant Cholesterol Inflammation Index; ADHF, acute decompensated heart failure.

A restricted cubic spline analysis with 4 knots was used to evaluate the dose-response relationship between LnRCII and 30-day mortality in ADHF patients. As shown in **Figure 3**, a marginally positive nonlinear association was observed between LnRCII and 30-day mortality (*P* for nonlinearity = 0.060). Notably, this nonlinear association exhibited an inverted L-shaped pattern, suggesting a potential inflection point where the nature of the association may change. When LnRCII was below this inflection point, the 30-day mortality risk remained relatively stable. However, when LnRCII exceeded the inflection point, the mortality risk progressively increased with higher LnRCII levels. To further quantify this threshold effect of LnRCII on 30-day mortality in ADHF patients, a two-piecewise Cox regression model was employed. As presented in **Table 3**, the inflection point (threshold) for LnRCII in relation to 30-day mortality was identified at 3.74. LnRCII < 3.74 may be associated with lower 30-day mortality in this cohort.

**Table 3:** The result of the two-piecewise Cox regression model.

Independent variable	Hazard ratios (95% confidence interval)	*P*-value	
The inflection point of LnRCII	3.74		
** < 3.74**	1.10 (0.81, 1.50)		0.555
** > 3.74**	1.99 (1.47, 2.69)	<0.001	
*P* for likelihood test	0.028		

RCII, remnant cholesterol inflammatory index.

### Exploratory subgroup analysis

**Figure 4:**
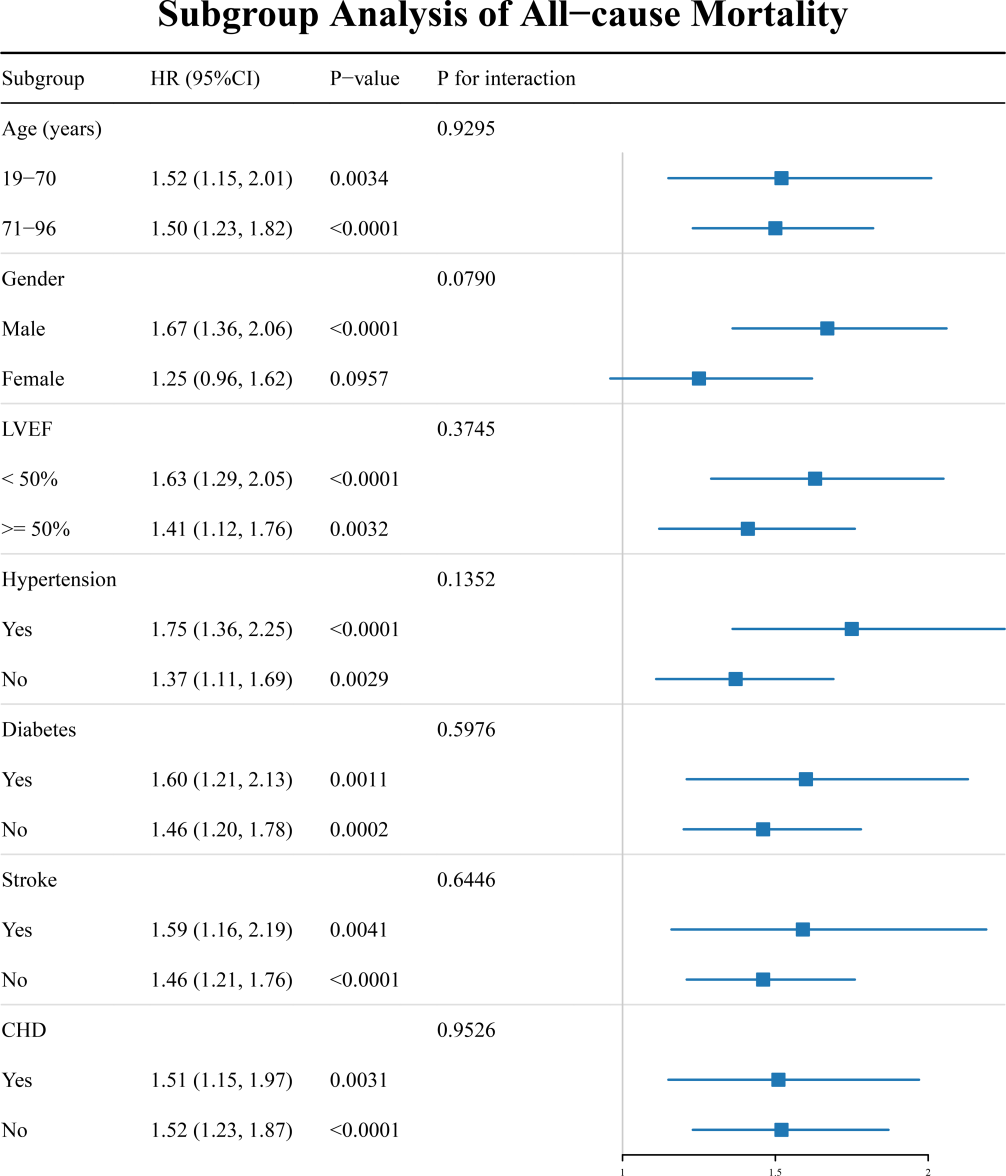
Forest plot of the association between RCII and all-cause mortality in subgroups. RCII, Remnant Cholesterol Inflammation Index; ADHF, acute decompensated heart failure.

To evaluate the potential heterogeneity in the association between LnRCII and 30-day mortality among ADHF patients, we conducted exploratory stratified analyses based on age, sex, LVEF, hypertension, diabetes, stroke, and CHD. The results are presented in **Figure 4**. In summary, LnRCII was significantly associated with an increased risk of 30-day mortality in most subgroups. It is noteworthy that a marginally significant interaction effect was observed in the sex subgroup (*P* for interaction = 0.079): a significant positive association was found between LnRCII and mortality risk in male patients (HR: 1.67, 95% CI: 1.36–2.06), whereas the association observed in female patients was not statistically significant (HR: 1.25, 95% CI: 0.96–2.07), suggesting a stronger association in males.

### Predictive performance of RCII versus RC and traditional lipid parameters (TG, TC, HDL-C, LDL-C)

**Figure 5:**
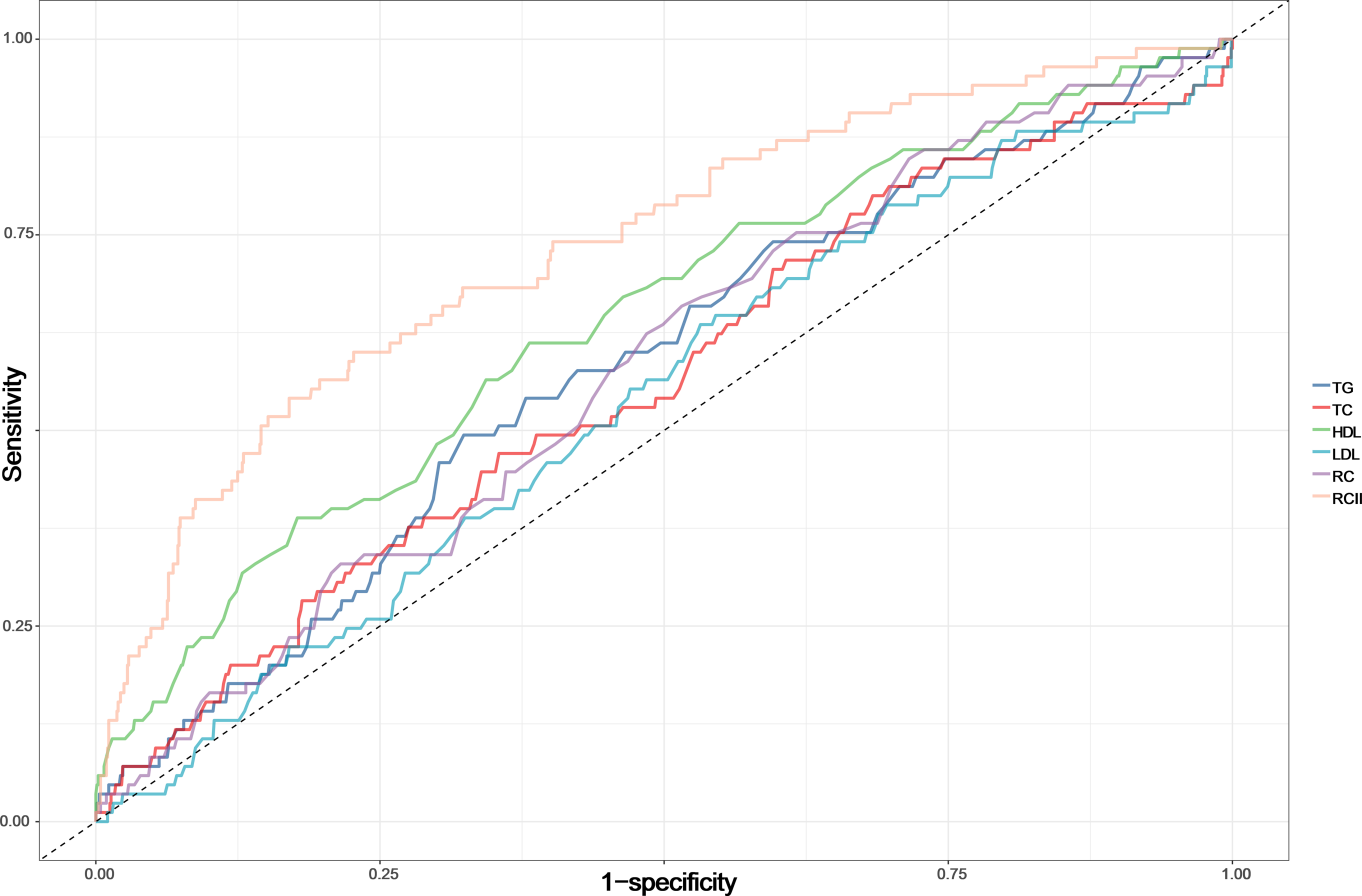
Receiver operating characteristic curves comparing RCII with RC or traditional lipid parameters (TG, TC, HDL-C, LDL-C) for predicting all-cause mortality in ADHF patients. RCII, Remnant Cholesterol Inflammation Index; ADHF, acute decompensated heart failure.

As shown in **Figure 5**, RCII demonstrated significantly superior predictive performance for 30-day mortality in ADHF patients compared with traditional lipid parameters and RC (AUCs: RCII 0.74, TG 0.58, TC 0.56, HDL-C 0.64, LDL-C 0.54, RC 0.58; all DeLong *p* < 0.01). The optimal cutoff value for LnRCII was calculated as 3.86 (**Table 4**).

**Table 4:** ROC curve analysis of the predictive value of RCII and conventional lipid parameters for 30-day mortality prognosis in ADHF patients.

Variable	AUC	95%CI low	95%CI upp	Best threshold	Specificity	Sensitivity
TG*	0.5817	0.5187	0.6448	1.3850	0.6763	0.4941
TC*	0.5629	0.4977	0.6281	4.2450	0.3165	0.8000
HDL-C*	0.6415	0.5782	0.7048	0.9050	0.6186	0.6118
LDL-C*	0.5400	0.4767	0.6032	2.2650	0.4680	0.6353
RC*	0.5780	0.5169	0.6391	16.4348	0.4845	0.6588
RCII	0.7373	0.6791	0.7955	47.6666	0.7732	0.6000
LnRCII	0.7373	0.6791	0.7955	3.8642	0.7732	0.6000

TG, triglyceride; TC, total cholesterol; HDL-C, high-density lipoprotein cholesterol; LDL-C, low-density lipid cholesterol; RC, remnant cholesterol; RCII, remnant cholesterol inflammatory index; ROC, receiver operating characteristic curve; AUC, area under the curve; CI, confidence interval.

*Delong *P* < 0.01, compare with RCII.

### Incremental predictive value of RCII for mortality risk assessment

Adding RCII to the baseline risk model significantly improved the predictive performance for 30-day mortality risk, demonstrating good incremental value (**Table 5**). Specifically, the C index of the model increased significantly from 0.85 to 0.87 (*P* < 0.01), and the IDI was 0.05 (*P* = 0.04). These findings indicate that incorporating RCII effectively enhances the risk stratification capability for short-term prognosis.

**Table 5:** Incremental predictive value of RCII for mortality risk assessment.

Model comparison	C-index	*P*-value	Integrated discrimination index (95% CI)	*P* value
Baseline risk model	0.85	–	–	–
Baseline risk model + RCII	0.87	<0.01	0.05 (0.01, 0.10)	0.04

RCII, remnant cholesterol inflammatory index; CI, confidence interval.

### Exploratory mediation analysis

A mediation analysis was applied to further investigate the potential mediating roles of GGT and ALB in the association between LnRCII and 30-day mortality in ADHF patients. The analysis results indicated that ALB exerted a significant partial mediating effect on this association. Specifically, the mediated proportion was approximately 11.45% (**Table 6**, *P*-value for proportion mediated = 0.012). It is noteworthy that the mediating effect of the oxidative stress marker GGT did not reach statistical significance.

**Table 6:** Mediated analysis was performed to explore the roles of GGT and ALB in the association between LnRCII and the 30-day mortality rate in ADHF patients.

Mediator	Total effect	Mediation effect	Direct effect	PM(%)	*P*-value of PM
ALB	0.017 (0.005, 0.030)	0.002 (0.001, 0.003)	0.015 (0.004, 0.028)	11.45	0.012
GGT	0.064 (0.044, 0.082)	0.001 (-0.000, 0.002)	0.064 (0.045, 0.083)	3.70	0.112

GGT, gamma-glutamyltransferase; ALB, albumin; PM, proportion mediate; ADHF, acute decompensated heart failure; other abbreviations as in **Table 1**.

The model adjusted for the same covariates as in model II (**Table 2**), except for the mediator variable.

### Sensitivity analysis

Several sensitivity analyses were performed, and the results were consistent with the primary findings (**Supplementary Tables 5–9**). Specifically, first, after excluding individuals with frailty, the results remained largely unchanged. Second, after excluding patients with concurrent pulmonary infections, the analysis results maintained consistency. Third, after excluding patients who received statin therapy at admission, the results of the re-analysis remained consistent with the primary findings. Fourth, after excluding patients who died within 48 hours after admission and repeating the primary analysis, no substantial changes were observed in the results. Finally, we constructed a complete dataset and repeated the primary analysis, with robust results.

## Discussion

In this retrospective cohort study conducted based on Jiangxi-ADHF II, we explored for the first time the association between RCII and 30-day all-cause mortality in patients with ADHF, and obtained the following key findings: First, there was a significant positive association between RCII and the risk of 30-day all-cause mortality. Second, RCII demonstrated superior predictive performance for 30-day all-cause mortality compared with traditional lipid parameters (including TG, TC, HDL-C, and LDL-C) and RC. In addition, the predictive performance was further improved after adding RCII to the baseline risk model. Collectively, these findings suggest that RCII holds promising clinical potential for early risk stratification in patients with ADHF following hospital admission.

RC refers to the cholesterol content remaining in the bloodstream after the partial lipolysis of TG-rich lipoproteins (23, 40). These remnants possess highly atherogenic properties; they can persist in the circulation, readily infiltrate and accumulate within the vascular intima, and drive the formation and progression of atherosclerotic plaques by inducing endothelial dysfunction and low-grade inflammatory responses (24, 25, 41, 42). Numerous previous clinical studies have confirmed that elevated RC levels are significantly associated with adverse outcomes in various CVDs (43–47). Meanwhile, the inflammatory response also plays a pivotal role in the pathological progression of atherosclerosis. Inflammation can induce endothelial dysfunction, promote the formation of lipid streaks, drive plaque progression and instability, and ultimately mediate the occurrence of a series of cardiovascular events (14, 48, 49). As a classic and extensively validated inflammatory marker, CRP is primarily synthesized by the liver in response to cytokines such as interleukin-6 and has been established as an important indicator in cardiovascular risk assessment (50, 51). A registry-based analysis of 3,831 Korean patients with acute HF revealed that patients with high CRP levels (Tertile 3 group) had a 302% higher risk of in-hospital mortality compared with those in the low-level group (Tertile 1 group) (52). A study by Zairis et al. of 577 ADHF patients demonstrated that patients with CRP levels > 3.8 mg/dL had a 60% higher risk of 31-day all-cause mortality compared with those with lower CRP levels (53). Similarly, a 120-day follow-up study of 4,777 Japanese patients hospitalized for acute HF conducted by Minami Y et al. showed that the highest CRP tertile (>11.8 mg/L) was significantly associated with an increased risk of all-cause mortality (HR = 2.21) (54). Furthermore, two studies from Japan and South Korea consistently confirmed the significant prognostic value of CRP in evaluating the long-term outcomes of patients with acute HF (55, 56).

It is important to emphasize that the lipid metabolism disorder represented by RC and the systemic inflammatory state reflected by CRP do not exist in isolation during the development of atherosclerosis but rather interact significantly and jointly promote the occurrence of adverse cardiovascular events. Multiple studies have indicated that in assessing cardiovascular event risk, a comprehensive evaluation combining RC and CRP demonstrates better predictive value than using either indicator alone (30–34). Based on the aforementioned mechanisms and clinical evidence, this study specifically focused on RCII, a novel index that combines RC and CRP. RCII was first proposed and its clinical value validated by Chen et al. in 2024 (37). Subsequently, based on two large longitudinal cohorts, the National Health and Nutrition Examination Survey and the China Health and Retirement Longitudinal Study, Wang et al. reported for the first time an inverted L-shaped association between LnRCII and all-cause mortality in the general population, identifying threshold effect points between 1.5–2 (in the Chinese population) and 0–1 (in the U.S. population), respectively (36). In the Jiangxi-ADHF II cohort of the present study, we observed a similar inverted L-shaped association between LnRCII and 30-day mortality. Using a two-piecewise Cox regression model, we identified the risk inflection point for RCII in this cohort as 3.74, which is slightly higher than the threshold reported in Wang et al.’s study. This discrepancy likely stems from the specific pathophysiological status of the study population. As is well known, ADHF is a complex clinical syndrome whose pathophysiological processes are often accompanied by neurohormonal activation, systemic inflammatory responses, insulin resistance, and visceral congestion (1, 57–59). Hepatic and intestinal congestion may directly interfere with the normal absorption, transport, and synthesis of cholesterol. Insulin resistance, meanwhile, promotes lipolysis, increases the delivery of free fatty acids to the liver, and consequently stimulates the production of very-low-density lipoprotein (VLDL) and chylomicrons (40, 60). Collectively, these ADHF-specific pathological alterations may contribute to an overall elevation in baseline RCII levels among patients. Compared with the study by Wang et al. based on general populations (Chinese cohort: LnRCII 0.82 ± 1.56; US cohort: LnRCII 1.71 ± 1.74), the average LnRCII level in our cohort was significantly higher (2.79 ± 1.65), which provides a reasonable clinical explanation for the observed difference in risk inflection point.

Exploratory subgroup analysis revealed a clinically meaningful finding: the association between LnRCII and 30-day mortality in ADHF patients may be sex-specific, with higher LnRCII levels correlating with an elevated risk of 30-day mortality in male patients. This is consistent with previous studies on sex differences in HF prognosis, which consistently indicate that male patients generally have a higher mortality risk (61–66). We therefore hypothesize that this sex-specific association may be explained by a combination of the following mechanisms: (1) Behavioral factors. Men tend to have higher rates of smoking and alcohol consumption (67). These factors can accelerate atherosclerosis by promoting inflammatory responses, oxidative stress, and endothelial dysfunction, among other pathways (68–71). In this context, the lipid-inflammatory pathway indicated by elevated RCII may act synergistically with these behavioral risk factors, further exacerbating atherosclerosis and thereby amplifying mortality risk. (2) Intrinsic sex differences in HF phenotypes and etiologies. Male patients more frequently present with HF with reduced ejection fraction, with CHD as the primary underlying cause. In contrast, females are more likely to have HF with preserved ejection fraction, with hypertension being a more common etiology (61–63). Consequently, in male patients, elevated RCII may interact with CHD-related atherosclerotic processes to promote the progression of coronary artery disease, exacerbate myocardial ischemia and hypoxia, and thereby exert a more pronounced adverse impact on prognosis. (3) Finally, the regulatory role of sex hormones cannot be overlooked. Under physiological conditions, androgens exhibit endothelial protective and anti-inflammatory properties, contributing to the maintenance of cardiovascular homeostasis (72–74). However, with advancing age, androgen levels in men gradually decline (75, 76), leading to a diminution of these cardioprotective effects.

### Clinical implications

The findings of this study hold important guiding significance for clinical practice. Compared with traditional biomarkers, RCII captures the synergistic pathogenic effects between atherogenic lipid disorders and inflammatory responses, thereby providing more comprehensive information for assessing cardiovascular event risk. Furthermore, RCII demonstrates high clinical utility and cost-effectiveness. This index can be measured through routine venous blood sampling and requires no specialized equipment or invasive procedures. Its ease of implementation facilitates widespread adoption across healthcare institutions at all levels, making it particularly suitable for widespread application in primary care settings with relatively limited medical resources.

### Limitations

Several limitations of this study should be acknowledged: First, its conduct within a single-center cohort in Jiangxi, China, may limit the generalizability of the findings to broader populations. Second, owing to the observational nature of the study, the association between RCII and short-term mortality in ADHF patients cannot be interpreted as causal. Therefore, our findings require validation in large-scale, multicenter prospective studies. Third, RCII was assessed at a single time point, precluding analysis of its dynamic changes during treatment, which might have yielded further prognostic insights. Fourth, despite adjusting for available confounders and conducting sensitivity analyses to support the robustness of our findings, the observational design cannot fully exclude residual confounding by unmeasured factors. Fifth, although the threshold value of LnRCII in the present study has certain clinical value, our findings are based on a single dataset and remain exploratory. External validation in future studies is therefore recommended. Finally, as this was an observational study, the mediation analysis is susceptible to residual confounding and violations of mediation assumptions (e.g., unmeasured confounding between RCII and ALB, and between ALB and mortality; ALB may be a marker of global illness severity rather than a true mediator), and thus cannot establish causality. The results are primarily intended to be hypothesis-generating and to provide directions for future mechanistic studies with more rigorous designs.

## Conclusion

In conclusion, this cohort study from Jiangxi, China, identified LnRCII as a novel and effective predictor of short-term mortality in patients with ADHF.

## Data Availability

The raw data supporting the conclusions of this article will be made available by the authors, without undue reservation.
