# Supplementary material for: Remnant cholesterol inflammation index as a predictor of mortality in patients with acute decompensated heart failure: evidence from the Jiangxi, China cohort
Source: Front Endocrinol (Lausanne). 2026 Apr 23;17:1792583. doi: 10.3389/fendo.2026.1792583 (PMC13149115; doi:10.3389/fendo.2026.1792583)
Supplement: Supplementary file 1 [file DataSheet1.docx]

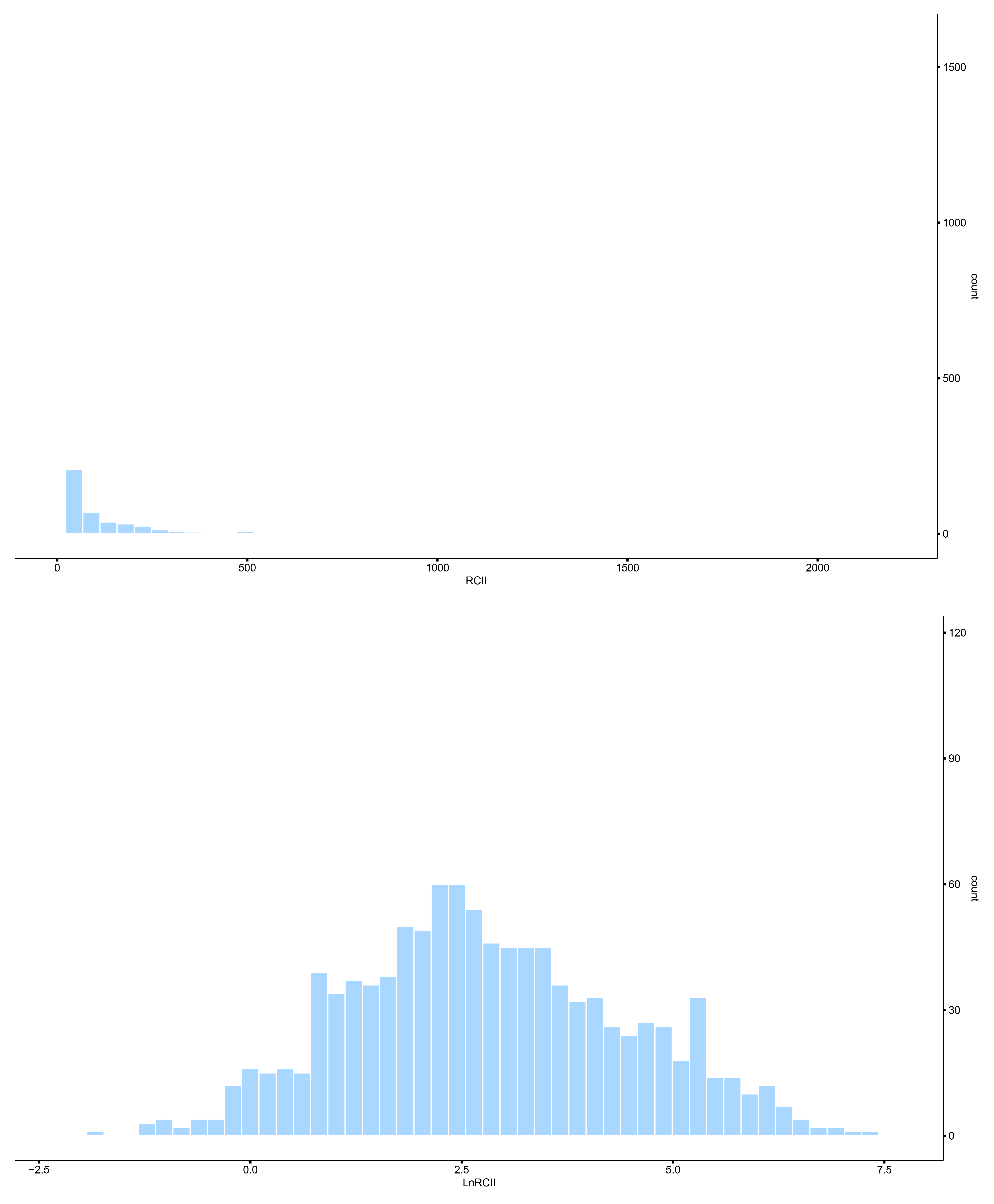


**Supplementary Figure 1:** Histogram distributions of RCII versus LnRCII.


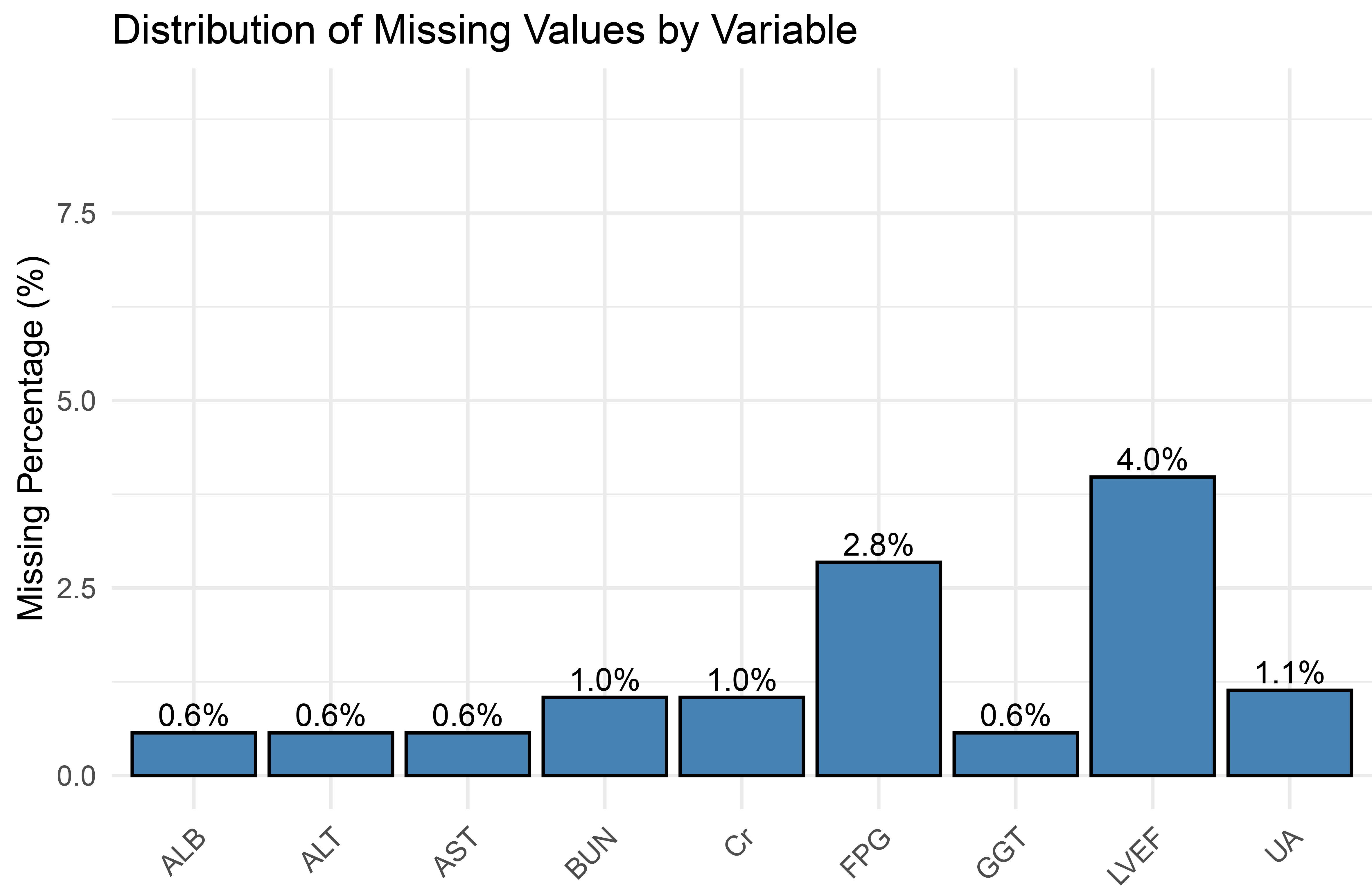


**Supplementary Figure 2:** Proportion of missing covariates.


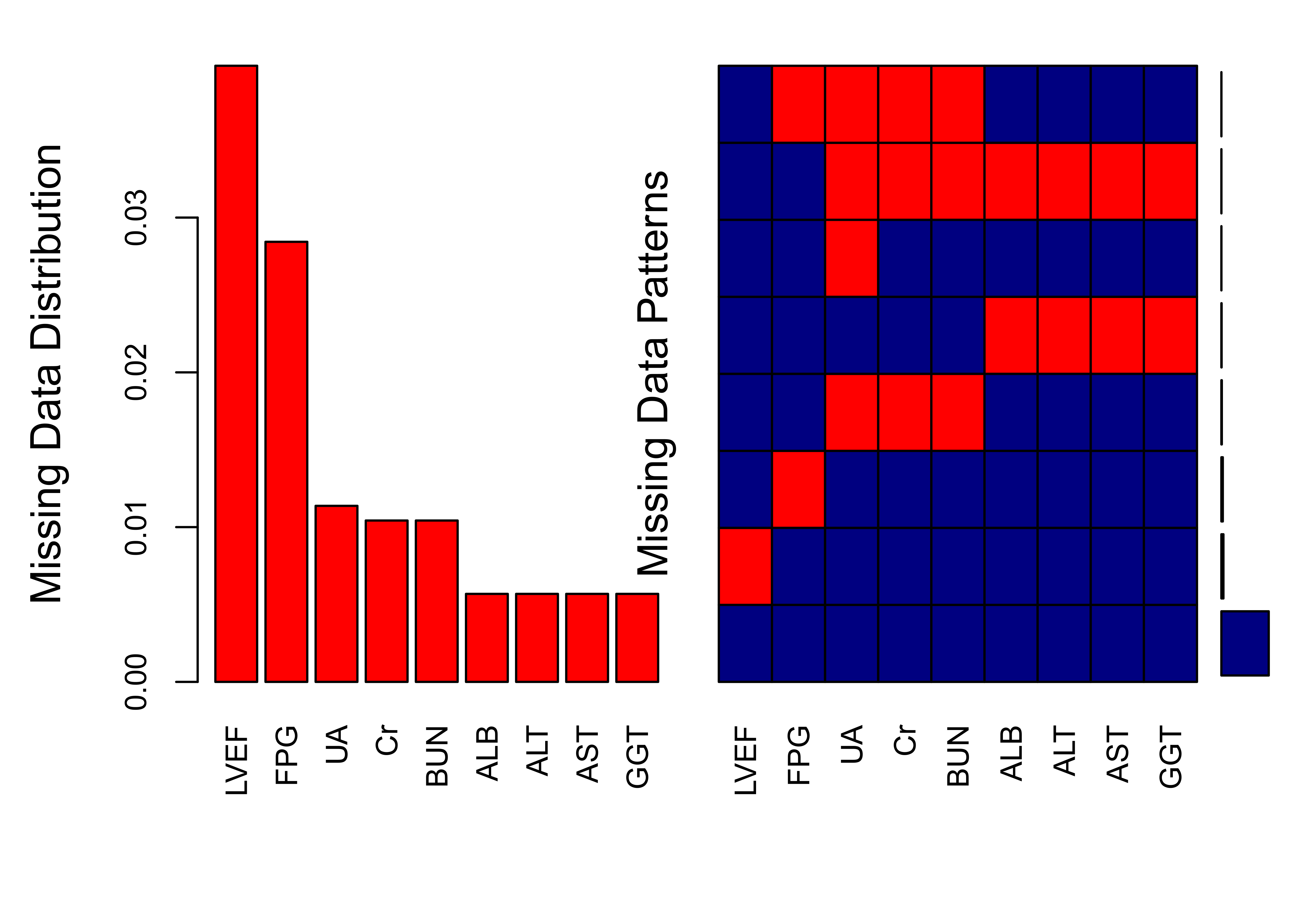


**Supplementary Figure 3:** Missing data cross-information diagram.


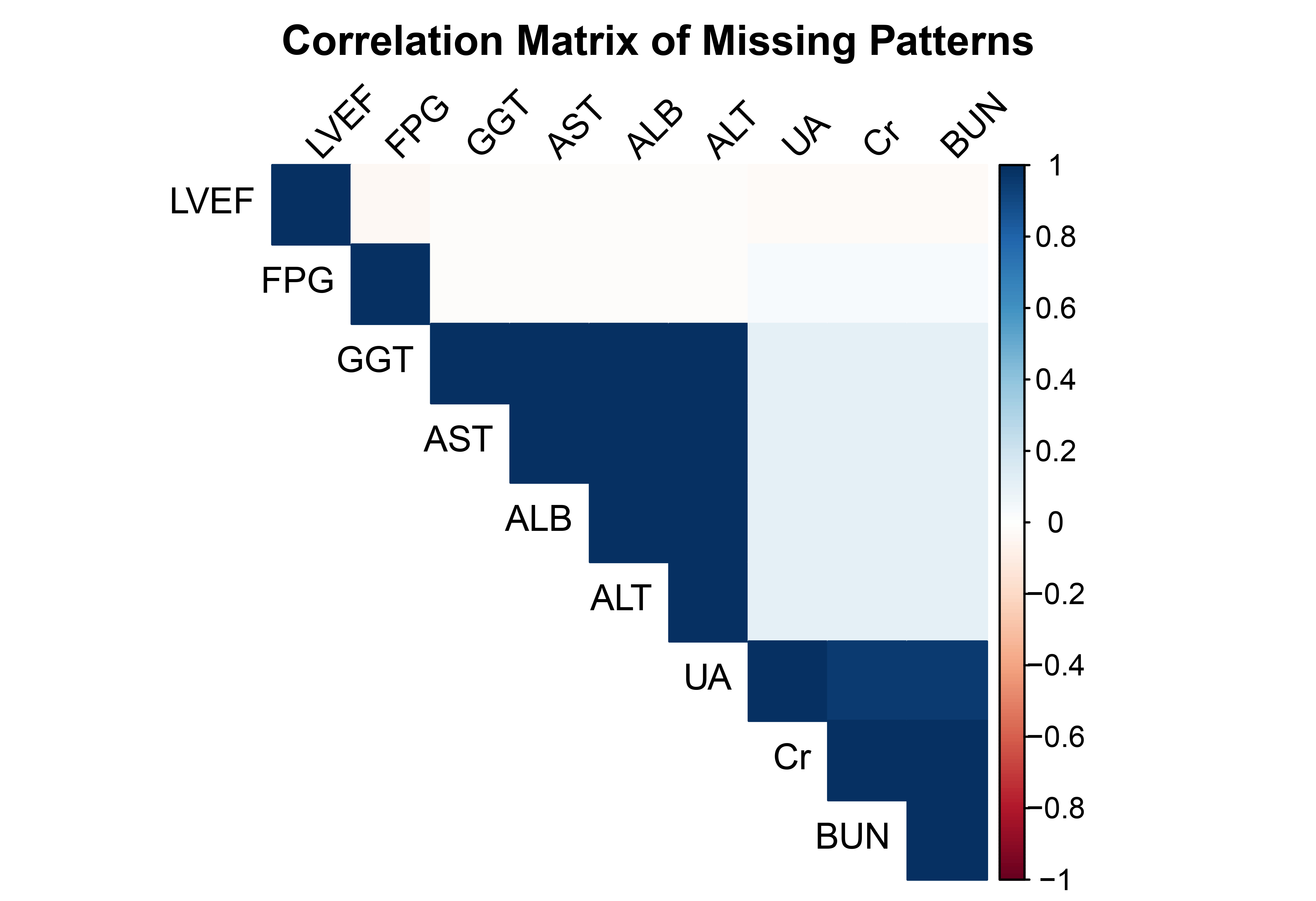


**Supplementary Figure 4**: Correlation matrix of missing data patterns.

Note: The correlation matrix shows relationships between missing patterns of variables. Missing values are encoded as 1, non-missing as 0, and pairwise correlations are calculated using the pairwise.complete.obs method. High values indicate strong dependencies in missingness.


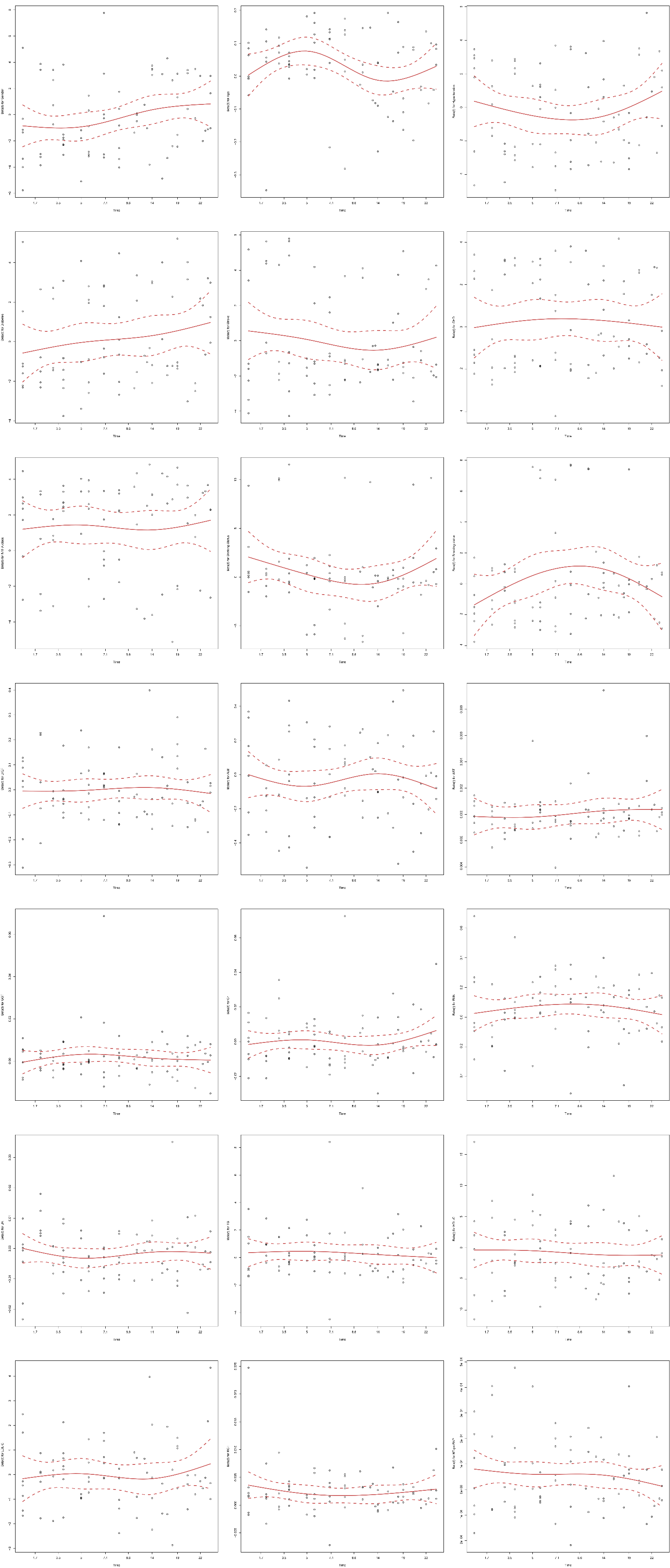


**Supplementary Figure 5:** Residual plot for validating the proportional hazards assumption.
