# Supplementary material for: Remnant cholesterol inflammation index as a predictor of mortality in patients with acute decompensated heart failure: evidence from the Jiangxi, China cohort
Source: Front Endocrinol (Lausanne). 2026 Apr 23;17:1792583. doi: 10.3389/fendo.2026.1792583 (PMC13149115; doi:10.3389/fendo.2026.1792583)
Supplement: Supplementary file 2 [file Table1.docx]

Supplementary Table 1: Comparing baseline characteristics of participants with and without missing RCII.

|  | RCII | | *P*-value |
| --- | --- | --- | --- |
|  | Missing | Non-Missing |  |
| No. of subjects | 1747 | 1055 |  |
| Age (years) | 70.00 (61.00-79.00) | 71.00 (60.00-80.00) | 0.440 |
| LVEF (%) | 45.00 (36.00-56.00) | 49.00 (39.00-56.00) | <0.001 |
| ALB (g/L) | 35.66 (4.97) | 34.83 (5.21) | <0.001 |
| ALT (U/L) | 21.00 (14.00-38.00) | 22.00 (14.00-39.00) | 0.997 |
| AST (U/L) | 26.00 (20.00-39.00) | 26.00 (20.00-40.00) | 0.561 |
| GGT (U/L) | 41.00 (25.00-73.00) | 44.00 (25.00-77.00) | 0.424 |
| Cr (umol/L) | 91.00 (71.00-126.00) | 88.00 (69.00-126.00) | 0.301 |
| BUN (mmol/L) | 7.60 (5.78-10.69) | 7.42 (5.60-10.68) | 0.353 |
| UA (umol/L) | 434.00 (346.00-545.00) | 420.00 (327.50-537.00) | 0.065 |
| TC (mg/dL) | 148.97 (40.01) | 148.42 (42.12) | 0.742 |
| TG (mg/dL) | 100.12 (76.20-134.67) | 101.00 (77.08-138.22) | 0.417 |
| HDL-C (mg/dL) | 37.89 (30.93-45.62) | 37.50 (30.35-45.62) | 0.735 |
| LDL-C (mg/dL) | 86.60 (67.27-109.02) | 85.05 (67.66-107.86) | 0.282 |
| RC (mg/dL) | 17.40 (10.44-26.29) | 17.01 (11.60-25.13) | 0.452 |
| CRP (mg/dL) | 1.03 (0.33-5.06) | 0.88 (0.37-2.80) | 0.070 |
| FPG (mmol/L) | 5.40 (4.70-6.30) | 5.40 (4.70-6.30) | 0.779 |
| NT-proBNP (pmol/L) | 3696.00 (1985.00-6098.50) | 3526.00 (1707.00-6399.50) | 0.350 |
| Gender |  |  | 0.995 |
| Male | 1012 (57.93%) | 611 (57.91%) |  |
| Female | 735 (42.07%) | 444 (42.09%) |  |
| NYHA classification (n,%) |  |  | 0.349 |
| III | 1176 (67.32%) | 692 (65.59%) |  |
| IV | 571 (32.68%) | 363 (34.41%) |  |
| Hypertension |  |  | 0.806 |
| No | 982 (56.21%) | 588 (55.73%) |  |
| Yes | 765 (43.79%) | 467 (44.27%) |  |
| Diabetes |  |  | 0.324 |
| No | 1311 (75.04%) | 774 (73.36%) |  |
| Yes | 436 (24.96%) | 281 (26.64%) |  |
| Stroke |  |  | 0.027 |
| No | 1480 (84.72%) | 860 (81.52%) |  |
| Yes | 267 (15.28%) | 195 (18.48%) |  |
| CHD |  |  | 0.234 |
| No | 1176 (67.32%) | 733 (69.48%) |  |
| Yes | 571 (32.68%) | 322 (30.52%) |  |
| Drinking status |  |  | 0.543 |
| No | 1581 (90.50%) | 962 (91.18%) |  |
| Yes | 166 (9.50%) | 93 (8.82%) |  |
| Smoking status |  |  | 0.930 |
| No | 1471 (84.20%) | 887 (84.08%) |  |
| Yes | 276 (15.80%) | 168 (15.92%) |  |
| 30-day mortality |  |  | 0.198 |
| No | 1629 (93.25%) | 970 (91.94%) |  |
| Yes | 118 (6.75%) | 85 (8.06%) |  |

Abbreviations as in Table 1.

Supplementary Table 2. The missing number and rate of covariates.

| Variables | Non- Missing | Missing |
| --- | --- | --- |
| Age | 1055 | 0 |
| Gender | 1055 | 0 |
| NYHA classification | 1055 | 0 |
| Hypertension | 1055 | 0 |
| Diabetes | 1055 | 0 |
| Stroke | 1055 | 0 |
| CHD | 1055 | 0 |
| Drinking status | 1055 | 0 |
| Smoking status | 1055 | 0 |
| LVEF | 1013 | 42 |
| ALB | 1049 | 6 |
| ALT | 1049 | 6 |
| AST | 1049 | 6 |
| GGT | 1049 | 6 |
| Cr | 1044 | 11 |
| BUN | 1044 | 11 |
| UA | 1043 | 12 |
| TC | 1055 | 0 |
| TG | 1055 | 0 |
| HDL-C | 1055 | 0 |
| LDL-C | 1055 | 0 |
| RC | 1055 | 0 |
| CRP | 1055 | 0 |
| RCII | 1055 | 0 |
| FPG | 1025 | 30 |
| NT-proBNP | 1055 | 0 |

Abbreviations as in Table 1.

Supplementary Table 3: Using all-cause mortality as the dependent variable, the proportional hazards assumption for covariates included in the model was tested via Schoenfeld residuals.

| Variable | chisq | df | *P*-value |
| --- | --- | --- | --- |
| Gender | 3.749 | 1 | 0.053 |
| Age | 1.727 | 1 | 0.189 |
| Hypertension | 0.009 | 1 | 0.924 |
| Diabetes | 3.414 | 1 | 0.065 |
| Stroke | 0.649 | 1 | 0.421 |
| CHD | 0.118 | 1 | 0.731 |
| NYHA class | 0.19 | 1 | 0.663 |
| Drinking Status | 0.335 | 1 | 0.563 |
| Smoking status | 0.052 | 1 | 0.819 |
| LVEF | 0.111 | 1 | 0.739 |
| ALB | 0.007 | 1 | 0.933 |
| AST | 0.787 | 1 | 0.375 |
| GGT | 0 | 1 | 0.985 |
| Cr | 0.831 | 1 | 0.362 |
| BUN | 0.242 | 1 | 0.623 |
| UA | 0.445 | 1 | 0.505 |
| TG | 0.016 | 1 | 0.900 |
| HDL-C | 0.147 | 1 | 0.702 |
| LDL-C | 0.057 | 1 | 0.812 |
| RCII | 0.256 | 1 | 0.613 |
| NT-proBNP | 0.496 | 1 | 0.481 |
| Global Schoenfeld Test p | 15.318 | 21 | 0.807 |

Abbreviations as in Table ​1.

Supplementary Table 4: Collinearity diagnostics steps.

|  | VIF | | | |
| --- | --- | --- | --- | --- |
|  | Step 1 | Step 2 | Step 3 | Step 4 |
| RCII | 4.2 | 4.2 | 4.2 | 1.4 |
| Age | 1.4 | 1.4 | 1.4 | 1.4 |
| Gender | 1.3 | 1.3 | 1.3 | 1.2 |
| NYHA classification | 1.2 | 1.2 | 1.2 | 1.2 |
| Hypertension | 1.2 | 1.2 | 1.2 | 1.2 |
| Diabetes | 1.4 | 1.4 | 1.4 | 1.4 |
| Stroke | 1.1 | 1.1 | 1.1 | 1.1 |
| CHD | 1.1 | 1.1 | 1.1 | 1.1 |
| Drinking status | 1.4 | 1.4 | 1.4 | 1.4 |
| Smoking status | 1.5 | 1.5 | 1.5 | 1.5 |
| LVEF | 1.3 | 1.3 | 1.3 | 1.2 |
| ALB | 1.3 | 1.3 | 1.3 | 1.3 |
| ALT | 6.4 | 6.4 | NA | NA |
| AST | 6.2 | 6.2 | 1.1 | 1.1 |
| GGT | 1.2 | 1.2 | 1.1 | 1.1 |
| Cr | 2.2 | 2.2 | 2.2 | 2.2 |
| BUN | 2.7 | 2.7 | 2.7 | 2.7 |
| UA | 1.6 | 1.6 | 1.6 | 1.6 |
| TC | 4760630847.5 | NA | NA | NA |
| TG | 2 | 2 | 2 | 2 |
| HDL-C | 375885739.9 | 1.4 | 1.4 | 1.4 |
| LDL-C | 2820298704.8 | 1.3 | 1.3 | 1.3 |
| RC | 723967991 | 2.6 | 2.6 | 2 |
| CRP | 3.4 | 3.4 | 3.4 | NA |
| FPG | 1.4 | 1.4 | 1.4 | 1.4 |
| NT-proBNP | 1.3 | 1.3 | 1.3 | 1.3 |

VIF: variance inflation factor; VIF = 1/(1-R^2^). Abbreviations as in Table ​1.

Note: The variables with VIF>3 will be regarded as collinear variables.

Supplementary Table 5 (Sensitivity-1): Multivariable Cox regression analysis of the association between RCII and 30-day mortality in patients with ADHF.

|  | Hazard ratios (95% confidence interval) | | | |
| --- | --- | --- | --- | --- |
|  | Unadjusted model | Model I | Model II | Model III |
| LnRCII | 1.73 (1.49, 2.01) | 1.71 (1.46, 1.99) | 1.59 (1.34, 1.89) | 1.53 (1.27, 1.84) |
| RCII (quartiles) |  |  |  |  |
| Q1 | Ref | Ref | Ref | Ref |
| Q2 | 2.28 (0.79, 6.55) | 2.23 (0.77, 6.42) | 1.46 (0.49, 4.31) | 0.80 (0.25, 2.52) |
| Q3 | 2.39 (0.83, 6.88) | 2.26 (0.78, 6.52) | 1.39 (0.47, 4.15) | 1.10 (0.36, 3.35) |
| Q4 | 9.50 (3.75, 24.04) | 8.55 (3.36, 21.80) | 5.31 (2.05, 13.76) | 3.54 (1.32, 9.50) |
| *P*-trend | <0.0001 | <0.0001 | <0.0001 | 0.0001 |

Abbreviations: ADHF: acute decompensated heart failure.

Model I adjusted for gender, age, drinking status, smoking status.

Model II adjusted for model I + hypertension, diabetes, stroke, CHD, NYHA classification, LVEF.

Model III adjusted for: Model II + ALB, AST, GGT, Cr, BUN, UA, NT-proBNP.

Supplementary Table 6 (Sensitivity-2): Multivariable Cox regression analysis of the association between RCII and 30-day mortality in patients with ADHF.

|  | Hazard ratios (95% confidence interval) | | | |
| --- | --- | --- | --- | --- |
|  | Unadjusted model | Model I | Model II | Model III |
| LnRCII | 2.21 (1.60, 3.06) | 2.27 (1.62, 3.16) | 2.25 (1.54, 3.30) | 2.59 (1.65, 4.05) |
| RCII (quartiles) |  |  |  |  |
| Q1 | Ref | Ref | Ref | Ref |
| Q2 | 5.41 (0.60, 48.38) | 5.17 (0.58, 46.34) | 4.02 (0.44, 36.61) | 2.75 (0.28, 27.09) |
| Q3 | 4.17 (0.43, 40.05) | 4.11 (0.43, 39.66) | 2.89 (0.29, 28.39) | 2.63 (0.26, 26.91) |
| Q4 | 27.47 (3.52, 214.58) | 28.22 (3.60, 221.50) | 20.82 (2.57, 168.66) | 21.24 (2.48, 181.93) |
| *P*-trend | 0.0001 | 0.0001 | 0.0011 | 0.0014 |

Abbreviations: ADHF: acute decompensated heart failure.

Model I adjusted for gender, age, drinking status, smoking status.

Model II adjusted for model I + hypertension, diabetes, stroke, CHD, NYHA classification, LVEF.

Model III adjusted for: Model II + ALB, AST, GGT, Cr, BUN, UA, NT-proBNP.

Supplementary Table 7 (Sensitivity-3): Multivariable Cox regression analysis of the association between RCII and 30-day mortality in patients with ADHF.

|  | Hazard ratios (95% confidence interval) | | | |
| --- | --- | --- | --- | --- |
|  | Unadjusted model | Model I | Model II | Model III |
| LnRCII | 1.78 (1.49, 2.13) | 1.72 (1.43, 2.08) | 1.70 (1.37, 2.11) | 1.44 (1.14, 1.83) |
| RCII (quartiles) |  |  |  |  |
| Q1 | Ref | Ref | Ref | Ref |
| Q2 | 2.09 (0.52, 8.36) | 1.89 (0.47, 7.60) | 1.37 (0.32, 5.78) | 0.76 (0.16, 3.51) |
| Q3 | 3.01 (0.80, 11.33) | 2.62 (0.69, 9.91) | 1.58 (0.39, 6.33) | 1.33 (0.33, 5.37) |
| Q4 | 10.74 (3.26, 35.41) | 8.73 (2.62, 29.10) | 6.41 (1.88, 21.91) | 3.17 (0.87, 11.51) |
| *P*-trend | <0.0001 | <0.0001 | 0.0001 | 0.0070 |

Abbreviations: ADHF: acute decompensated heart failure.

Model I adjusted for gender, age, drinking status, smoking status.

Model II adjusted for model I + hypertension, diabetes, stroke, CHD, NYHA classification, LVEF.

Model III adjusted for: Model II + ALB, AST, GGT, Cr, BUN, UA, NT-proBNP.

Supplementary Table 8 (Sensitivity-4): Multivariable Cox regression analysis of the association between RCII and 30-day mortality in patients with ADHF.

|  | Hazard ratios (95% confidence interval) | | | |
| --- | --- | --- | --- | --- |
|  | Unadjusted model | Model I | Model II | Model III |
| LnRCII | 1.74 (1.51, 2.02) | 1.72 (1.48, 2.00) | 1.63 (1.39, 1.91) | 1.52 (1.28, 1.81) |
| RCII (quartiles) |  |  |  |  |
| Q1 | Ref | Ref | Ref | Ref |
| Q2 | 2.81 (0.89, 8.82) | 2.74 (0.87, 8.61) | 2.24 (0.71, 7.06) | 1.38 (0.42, 4.52) |
| Q3 | 4.30 (1.45, 12.79) | 4.09 (1.38, 12.17) | 2.74 (0.91, 8.27) | 1.72 (0.56, 5.33) |
| Q4 | 11.87 (4.26, 33.06) | 10.63 (3.79, 29.77) | 7.63 (2.70, 21.57) | 4.63 (1.61, 13.30) |
| *P*-trend | <0.0001 | <0.0001 | <0.0001 | <0.0001 |

Abbreviations: ADHF: acute decompensated heart failure.

Model I adjusted for gender, age, drinking status, smoking status.

Model II adjusted for model I + hypertension, diabetes, stroke, CHD, NYHA classification, LVEF.

Model III adjusted for: Model II + ALB, AST, GGT, Cr, BUN, UA, NT-proBNP.

Supplementary Table 9 (Sensitivity-5): Multivariable Cox regression analysis of the association between RCII and 30-day mortality in patients with ADHF.

|  | Hazard ratios (95% confidence interval) | | | |
| --- | --- | --- | --- | --- |
|  | Unadjusted model | Model I | Model II | Model III |
| LnRCII | 1.72 (1.50, 1.97) | 1.70 (1.48, 1.95) | 1.63 (1.40, 1.90) | 1.53 (1.31, 1.79) |
| RCII (quartiles) |  |  |  |  |
| Q1 | Ref | Ref | Ref | Ref |
| Q2 | 2.21 (0.84, 5.81) | 2.14 (0.81, 5.63) | 1.62 (0.61, 4.32) | 1.10 (0.40, 2.98) |
| Q3 | 2.86 (1.13, 7.26) | 2.70 (1.06, 6.85) | 1.80 (0.70, 4.65) | 1.20 (0.46, 3.16) |
| Q4 | 8.91 (3.82, 20.80) | 7.92 (3.37, 18.59) | 5.58 (2.35, 13.27) | 3.78 (1.57, 9.07) |
| *P*-trend | <0.0001 | <0.0001 | <0.0001 | <0.0001 |

Abbreviations: ADHF: acute decompensated heart failure.

Model I adjusted for gender, age, drinking status, smoking status.

Model II adjusted for model I + hypertension, diabetes, stroke, CHD, NYHA classification, LVEF.

Model III adjusted for: Model II + ALB, AST, GGT, Cr, BUN, UA, NT-proBNP.
